# Bioorthogonal two-component drug delivery in HER2(+) breast cancer mouse models

**DOI:** 10.1038/srep24298

**Published:** 2016-04-12

**Authors:** Sudath Hapuarachchige, Yoshinori Kato, Dmitri Artemov

**Affiliations:** 1 Division of Cancer Imaging Research, Department of Radiology and Radiological Science, The Johns Hopkins University School of Medicine, Baltimore, MD 21205, USA; 2 Department of Oncology, The Sidney Kimmel Comprehensive Cancer Center, The Johns Hopkins University School of Medicine, Baltimore, MD 21205, USA; 3 Hoshi University School of Pharmacy and Pharmaceutical Sciences, Life Science Tokyo Advanced Research Center (L-StaR), Shinagawa-ku, Tokyo 142-8501, JAPAN

## Abstract

The HER2 receptor is overexpressed in approximately 20% of breast cancers and is associated with tumorigenesis, metastasis, and a poor prognosis. Trastuzumab is a first-line targeted drug used against HER2(+) breast cancers; however, at least 50% of HER2(+) tumors develop resistance to trastuzumab. To treat these patients, trastuzumab-based antibody-drug conjugates (ACDs) have been developed and are currently used in the clinic. Despite their high efficacy, the long circulation half-life and non-specific binding of cytotoxic ADCs can result in systemic toxicity. In addition, standard ADCs do not provide an image-guided mode of administration. Here, we have developed a two-component, two-step, pre-targeting drug delivery system integrated with image guidance to circumvent these issues. In this strategy, HER2 receptors are pre-labeled with a functionalized trastuzumab antibody followed by the delivery of drug-loaded nanocarriers. Both components are cross-linked by multiple bioorthogonal click reactions *in situ* on the surface of the target cell and internalized as nanoclusters. We have explored the efficacy of this delivery strategy in HER2(+) human breast cancer models. Our therapeutic study confirms the high therapeutic efficacy of the new delivery system, with no significant toxicity.

The HER2 receptor is one of four human plasma membrane receptors from the ErbB tyrosine kinase receptor family^1^. HER2 regulates the cellular proliferation and survival of cells by dimerizing with other ErbB receptors and stimulating tyrosine kinase activity^2^,^3^. Approximately 20–30% of human breast cancers overexpress HER2 receptors by amplification of the HER2/*neu* gene, which is a marker of aggressive cancer with an unfavorable prognosis that correlates with tumorigenesis and metastasis^4^,^5^,^6^,^7^. The anti-HER2 humanized monoclonal antibody, trastuzumab (Tz), is a first-line biotherapeutic against HER2(+) breast cancer^8^,^9^. However, recent clinical statistics have revealed that the long-term use of trastuzumab can generate trastuzumab resistance in HER2(+) tumors^10^. The causes of trastuzumab resistance are not yet fully understood^10^,^11^,^12^. The HER2 receptor also exhibits a poor ability to internalize, even after the binding of bioligands, such as anti-HER2 antibodies, HER2-specific antibody fragments, aptamers, and peptides^13^. Poor internalization of HER2 is also a potential drawback to the use of this receptor as a therapeutic target. To overcome the trastuzumab resistance in HER2(+) tumors, trastuzumab-based antibody-drug conjugates (ADCs), such as trastuzumab-emtansine (T-DM1), have been developed with the chemotherapeutic drug mertansine directly attached to the antibody, which boosts the cell toxicity. After successful clinical trials, T-DM1 is currently used in the clinic^14^,^15^. However, by design, ADCs are intrinsically highly toxic and can produce severe side effects due to their long circulatory half-life and non-specific toxicity in healthy tissues^16^. Furthermore, a simple ADC does not provide a mechanism by which to enhance the cellular internalization of therapeutics to maintain a high therapeutic index^17^.

To circumvent these issues, we have designed a pre-targeting two-component, two-step drug delivery system driven by bioorthogonal click chemistry between the pre-targeting and delivery components. In this strategy, HER2(+) cancer cells are pre-labeled by click-reactive trastuzumab, and subsequently, click-reactive, drug-loaded albumin nanocarriers (Alb) are delivered to enhance the internalization of drug carriers after the *in situ* bioorthogonal cross-linking of components, as shown in Fig. 1. The pre-targeting approach was used for imaging and therapy in lung cancer^18^,^19^,^20^. We have also demonstrated the efficacy of HER2 pre-targeted therapy in isolated cells^21^. However, to the best of our knowledge, this is the first demonstration of an anti-HER2 pre-targeted therapeutic strategy *in vivo*.

By definition, bioorthogonal reactions are reactions that can occur in living systems at physiological conditions without interfering with regular biochemical and physiological processes. For example, the Staudinger ligation, the copper-free azide/alkyne click reaction, and the *trans*-cyclooctene/tetrazine cycloaddition have been explored for *in vitro* and *in vivo* imaging. Azide (Az)/difluorocyclooctene (DIFO) click chemistry has been used by Bertozzi’s group for *in vivo* imaging in living systems^22^. Kim’s group has demonstrated the *in vivo* imaging of mouse tumor models using Az and dibenzylcyclooctyne (DBCO) click chemistry between metabolically labeled glycans and liposomes^18^. *Trans*-cyclooctene (TCO)/tetrazine (Tt) cycloadditions have been extensively studied for *in vivo* imaging experiments because this reaction is extremely fast (3,100–380,000,000 × 10^3^ M^−1^s^−1^) compared to the Az/DBCO (0.9–4,000 × 10^3^ M^−1^s^−1^) and Az/DIFO (7.6 × 10^−2^ M^−1^s^−1^) bioorthogonal click reactions^23^,^24^. The TCO/Tt click reaction has been used to image nanoparticles in living systems^25^. We have previously used Az/DBCO bioorthogonal click chemistry in two-component drug delivery system to evaluate the strategy *in vitro* in HER2(+) BT-474 cells^21^. We observed the cluster formation and internalization of nanoclusters with confocal fluorescence imaging. Our *in vitro* therapeutic experiments confirmed the high therapeutic efficacy of a two-component, two-step drug delivery system. Due to its fast kinetics, TCO/Tt cycloaddition was used in this study for the *in situ* conjugation of the components of a two-component, two-step drug delivery system in a HER2(+) human breast cancer mouse model, as shown in Fig. 1. In this strategy, due to the close proximity of overexpressed HER2 receptors on cancer cells and multiple functionalizations of the pre-targeting and delivery components, multiple cross-linking reactions induce the self-assembling of cell membrane-bound nanoclusters *in situ*. These nanoclusters can be effectively internalized by clathrin-mediated endocytosis^26^. In the present study, we employed the new delivery system in mouse models of HER2(+) human breast cancer, evaluated the strategy, and determined whether this system could enhance therapeutic efficacy.

## Results

### Enhancement of cellular internalization by a two-component delivery strategy

**Figure 2:**
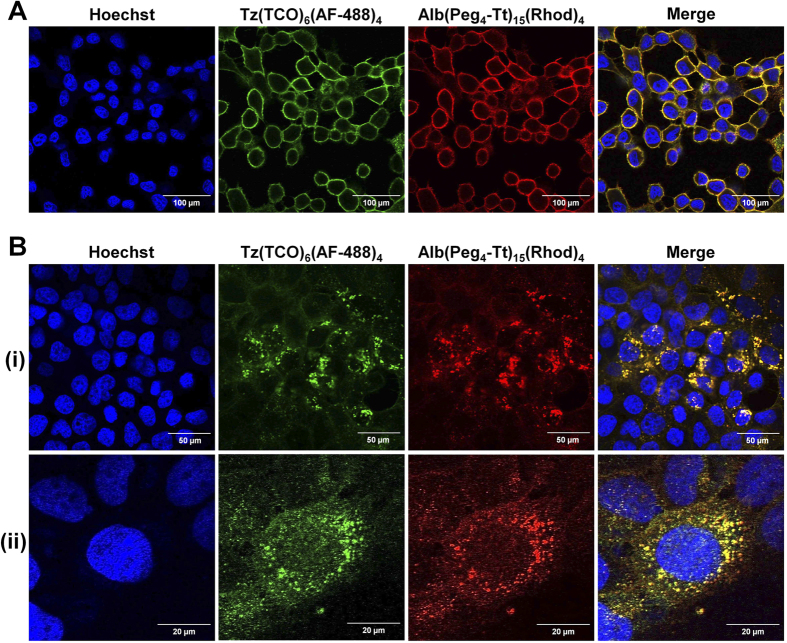
Validation of the internalization strategy by *in vitro* confocal microscopy. *In vitro* confocal fluorescent images of the two-component delivery strategy in HER2(+) BT-474 cells showing (**A**) cell surface labeling by Tz(TCO)_6_(AF-488)_4_ followed by the co-localization of Alb(Peg_4_-Tt)_15_(Rhod)_4_ after 15 min incubation at 20 °C (Scale bar: 100 μm), and (**B**) rapid cellular internalization after incubation at 37 °C for 4 h (Scale bar: 50 μm). (i) Full field-of-view 63x confocal images, and (ii) a zoomed-in, high-resolution section of the images (Scale bar: 20 μm).

The cellular internalization of components by the two-component strategy was evaluated in HER2(+) BT-474 breast cancer cells, using a confocal fluorescence microscope (Supplementary Figure S1A). As shown in Fig. 2A, the cell surface was initially labeled by a Tz(TCO)_6_(AF-488)_4_ pre-targeting component. Co-localization of the delivery component, Alb(Peg_4_-Tt)_15_(Rhod)_4_, with the pre-targeting components on the cell surface was detected immediately within 15 minutes after labeling. Upon incubation at 37 °C for 4 h, we observed a rapid internalization of co-localized nanoclusters formed by the two components (Fig. 2B). No cellular internalization was detected after incubation at 20 °C (Fig. 2A). Moreover, neither co-localization nor internalization of components was observed when reactive Tz(TCO)_6_(AF-488)_2_ and non-reactive Alb(Rhod)_4_ were used as the pre-targeting and delivery components, respectively (Supplementary Figure S2).

### Enhancement of tumor uptake of components

**Figure 3:**
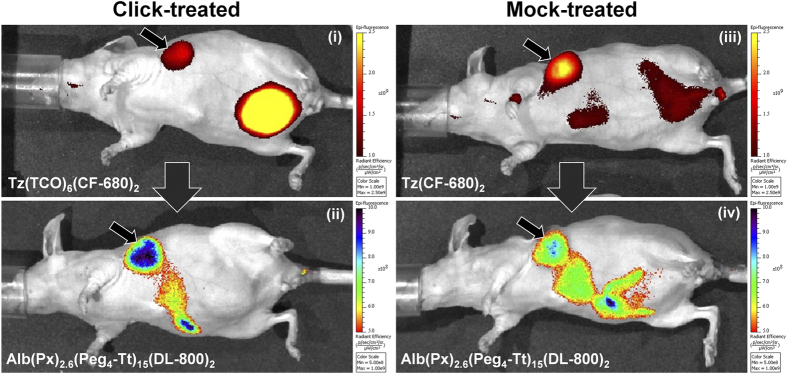
*In vivo* fluorescence imaging of drug delivery. *In vivo* Xenogen fluorescence optical images of the two-component, two-step delivery system measured at 8 h post-injection of the drug delivery component (20 h post-injection of the pre-targeting component). (i) Distribution of the functionalized pre-targeting component Tz(TCO)_6_(CF-680)_2_, and (ii) tumor uptake of the drug delivery component Alb(Px)_2.6_(Peg_4_-Tt)_15_(DL-800)_2_ in a click-treated mouse. (iii) Distribution of the non-reactive pre-targeting components, Tz(CF-680)_2_, and (iv) Alb(Px)_2.6_(Peg_4_-Tt)_15_(DL-800)_2_ in a mock-treated mouse.

We evaluated the tumor uptake and plasma clearance of circulating non-specifically bound components on an *in vivo* Xenogen optical imaging system (Supplementary Figure S1B). Three groups of mice, including click-treated, mock-treated, and untreated-controls were used in this study. The first two treatment groups, click-treated and mock-treated, were administered pre-targeting components, including a reactive Tz(TCO)_6_(CF-680)_2_ (where CF-680 is an NIR fluorophore), and a non-reactive Tz(CF-680)_2_, respectively. Animals were imaged for 12 h to measure the biodistribution of pre-targeting components. Generally, a rapid clearance of both components from the circulation was detected in our models. A significant accumulation of TCO-functionalized and non-functionalized pre-targeting components was detected in the tumors at approximately eight hours. For TCO-functionalized Tz, the degree of functionalization (DOF) of 6 was maintained. There was no significant change in the binding affinity of Tz with HER2 at DOF = 6 (Supplementary Figure S3)^27^. At this time-point, plasma was free of excess pre-targeting components; however, some amount of the component accumulated in the kidneys (Supplementary Figure S4A) and was found in urine, as well. After 24 h, the amount of pre-targeting component in the kidneys had decreased significantly and was completely cleared from the urine (Supplementary Figure S4B). At the eight-hour post-injection, the Alb(Px)_2.6_(Peg_4_-Tt)_15_(DL-800)_2_ therapeutic carrier was administered to the click-treated and mock-treated groups, where Px and DL-800 were paclitaxel and DyLight 800 NIR fluorophore, respectively, while the untreated group received saline. Animals were imaged to track the drug delivery component routinely for two days. During this imaging time-frame, pre-targeting components were still visible in tumors in both click-treated (Fig. 3-i, intensity 1,174 a.u.) and mock-treated (Fig. 3-iii, intensity 1,362 a.u.) mice, and mice in the click-treated group showed a higher tumor uptake of the Alb drug delivery component (Fig. 3-ii, intensity 6,753 a.u.) compared to mice in the mock-treated group (Fig. 3-iv, intensity 3,327 a.u.).

### High cellular internalization of components *in vivo*

**Figure 4:**
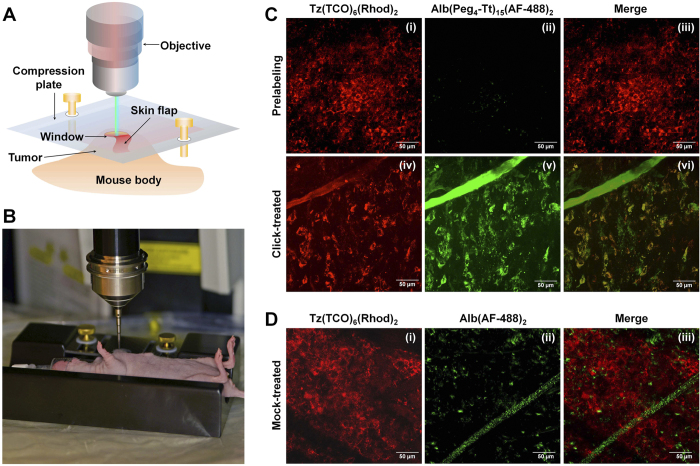
Evaluation of the pre-targeting approach by intravital microscopy. (**A**) Schematic view of the intravital microscopic imaging after skin-flap surgery. (**B**) Custom-made mouse-holder and set-up for intravital microscopy of cancer in mouse models. (**C**) Intravital multiphoton fluorescence images after the minimally invasive skin-flap surgery in click-treated mice (Scale bar: 50 μm). (i) Tumor uptake of pre-targeting Tz(TCO)_6_(AF-488)_2_ in the red channel after 12 h post-injection; (ii) autofluorescence in the green channel; (iii) merging two channels; (iv) tumor uptake of Tz(TCO)_6_(Rhod)_2_ after 1.5 h of Alb(Peg_4_-Tt)_15_(AF-488)_2_ injection; (v) tumor uptake of the Alb(Peg_4_-Tt)_15_(AF-488)_2_ delivery component; and (vi) merging of red and green channels shows the co-localization of the two components. (**D**) Intravital fluorescence images of the control mock therapy. (i) Tumor uptake of pre-targeting Tz(TCO)_6_(AF-488)_2_, (ii) control non-reactive Alb(AF-488)_2_, and (iii) merged images after 1.5 h of Alb(AF-488)_2_ injection.

To explore the *in vivo* cell labeling and cellular internalization of components, we observed the tumor microenvironment using an intravital multiphoton microscope during the treatment (Supplementary Figure S1C). Mice were first administered Tz(TCO)_2_(Rhod)_2_ intravenously, and, at eight-twelve hours post-injection, they were imaged after minimally invasive skin-flap surgery (Fig. 4A,B). The surface labeling of cancer cells by pre-targeting components was observed after 10 minutes and did not change significantly for twelve hours (Fig. 4C-i). At the next step, mice were administered Alb(Peg_4_-Tt)_15_(AF-488)_2_ intravenously and the imaging was continued for approximately two hours post-injection. The delivery of Alb(Peg_4_-Tt)_15_(AF-488)_2_ and the build-up of the fluorescence signal was detected and the signal was co-localized with the pre-targeting agent, Tz(TCO)_6_(Rhod)_2_ (Fig. 4C-v). No significant auto-fluorescence was detected in the green channel used to image the drug carrier component (Fig. 4C-ii). The co-localization of components and the internalization of clusters was observed starting at 30 min post-injection, and reached a maximal level after 90 minutes (Fig. 4C-vi). We observed neither co-localization nor internalized nanoclusters in the intravital imaging experiment repeated with reactive Tz(TCO)_6_(Rhod)_2_ (Fig. 4D-i) and non-reactive Alb(AF-488)_2_ (Fig. 4D-ii).

### Enhancement of therapeutic efficacy with low toxicological effects

**Figure 5:**
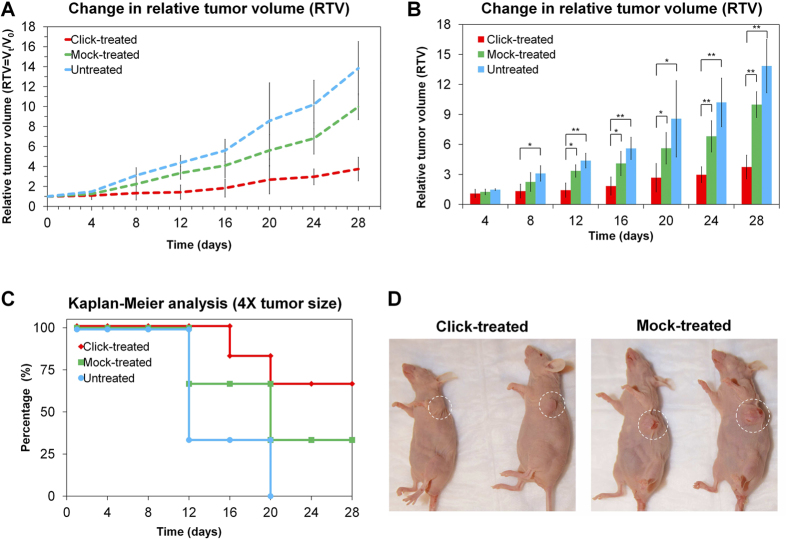
Therapeutic effects of the pre-targeted therapy. (**A**) Changes in relative tumor volumes (RTV = V_t_/V_0_) after two doses of treatment over 28 days in mouse models of BT-474 human breast cancer. (**B**) Statistical analysis of change in relative tumor volume (*p < 0.05, **p < 0.005). (**C**) The Kaplan-Meier analysis of surrogate survival using the time taken to reach four times the initial tumor size (**D**) Change in tumor appearance in mice after specific click-mediated therapy (left) and mock therapy with non-reactive components (right).

We evaluated the therapeutic effect based on the change in relative tumor volumes (RTV = tumor volume at day *t*, V_t_/V_0_, initial tumor volume), calculated from the tumor dimensions and measured by a caliper, over 28 days, with two doses of treatments (Supplementary Figure S1D). Mice in the click-treated and mock-treated groups were injected with Tz(TCO)_6_(CF-680)_2_ and Tz(CF-680)_2_, respectively, and the untreated control group received saline. After eight hours, the first two groups were administered Alb(Px)_2.6_(Peg_4_-Tt)_15_(CF-750)_2_ and the mice in the untreated-control group received saline. Mice received the second dose of therapy after 14 days. During the treatment period, the changes in tumor volumes and body weights were examined for 28 days and plotted as a function of time post-injection. The mice in the click-treated group exhibited a significant inhibition of tumor growth, as confirmed by the lowest RTV over the treatment period (Fig. 5A), compared to the mock-treated and untreated-control groups. The corresponding bar chart was used for the statistical analysis of the changes in relative tumor volumes (Fig. 5B). The relative tumor volume in the click-treated group was significantly low compared to the untreated group from day 8 onward (Fig. 5B), and it was significantly low compared to the mock-treated group after day 12 onward. The Kaplan-Meier analysis curves shown in Fig. 5C were obtained using changes in the terminal RTV by a factor of four from the initial tumor size. The highest RTV was detected in the untreated-control group. Visible changes in tumor sizes in different treatment groups are shown in Fig. 5D.

We also performed a comprehensive study of the toxicological effects in mouse models of BT-474 breast cancer. Mice in the click-treated and mock-treated groups received both components at doses of 0×, 1×, 2×, and 5× following the same treatment schedule used for the therapy (Supplementary Figure S1D). Based on the representative results of the study, there was no body weight loss (Supplementary Figure S5), and only mild thrombocytopenia (Supplementary Figure S6) was detected at 24 h post-treatment dose in the high-dose (5×) treatment group. However, in this group, the platelet count recovered by factor of five at day 28 of the therapy.

## Discussion

We chose trastuzumab as the target-specific pre-targeting ligand for HER2, since Tz has high target-specificity and binding affinity to HER2 receptors, even after chemical conjugations on its free amine groups^27^. Furthermore, Tz resistance in HER2(+) cancer cells does not decrease the degree of HER2 expression^28^. There is no HER2-specific endogenous ligand in the human body for competitive binding affinity with Tz. As a chemotherapeutic, Px has high therapeutic efficacy against solid tumors and can be easily modified and conjugated with drug delivery platforms without altering cytotoxicity^29^. Paclitaxel albumin conjugates are also used in chemotherapeutic regimens (Abraxan)^30^. Serum albumin was chosen as the drug carrier for several reasons. Albumin is an acidic, hydrophilic, and highly stable globular protein. It is stable in a broad pH range (pH 4–9), in 40% ethanol, and at high temperatures (60 °C) without denaturing^31^. Hydrophobic low-molecular-weight chemotherapeutics can be chemically conjugated with albumin without a significant change in the hydrophilicity of albumin in plasma. Drugs encapsulated in albumin exhibit favorable pharmacokinetics with albumin as a drug carrier. Albumin can also be accumulated in solid tumors by the enhanced permeability and retention (EPR) effect; however, our approach was intended to increase the cellular uptake of drug-loaded nanocarriers rather than to enhance the accumulation of nanocarriers in the tumor extracellular microenvironment^32^.

To synthesize drug-loaded nanocarriers, paclitaxel was first derivatized into an amine reactive *sulfo*-NHS analogue (*sulfo*-NHS-paclitaxel), and conjugated with albumin. The conjugation ratio of paclitaxel to albumin is a critical parameter that significantly decreases the hydrophilicity of the delivery component. Thus, the degree of conjugation (DOC) of paclitaxel was chosen at ~2.6 to synthesize the Alb(Px)_2.6_ precursor. Paclitaxel was conjugated with albumin by an ester linkage, which is stable *in vivo* and enables the efficient release of drugs after internalization, followed by acidic or enzymatic cleavage.

We evaluated the cell surface labeling and the internalization of nanoclusters *in vitro* by confocal fluorescence imaging. The poor internalization kinetics of Tz in HER2(+) cells increases its availability as a pre-targeting component on the cell surface for multiple click reactions with Alb-based delivery components. Multiple bioorthogonal click reactions in TCO/Tt-based delivery strategy were faster than those for previously used with Az/DBCO system^21^. The co-localization of two components on the cell surface provides efficient click reactions in physiological conditions. We also observed the formation of nanoclusters and their internalization after four hours incubation at 37 °C. The control Alb(Rhod)_4_ delivery component, which lacked functional Tt groups, did not react with the pre-targeting component and none of the components were internalized upon incubation (Supplementary Figure S2).

We explored the tumor uptake of components based on the *in vivo* fluorescent intensities of the components. Images acquired by Xenogen IVIS optical imaging suggested a high accumulation of pre-targeting Tz components in the tumor. Interestingly, we observed higher tumor uptake of non-reactive Tz(CF-680)_2_ in the mock-treated mice compared to the reactive Tz(TCO)_6_(CF-680)_2_ in the click-treatment. We suggest that this observation is, in part, due to the slight decrease in the binding affinity of trastuzumab functionalized with TCO (Supplementary Figure S3). In addition, specific click reactions between the components resulted in the formation of cross-linked complexes, which may have further reduced fluorescence by quenching. Finally, cell internalization of the complexes resulted in their fast degradation and clearance of the fluorescent marker observed in the bladder of the click-treated mice (Fig. 3-i).

Clinically, a relatively long mean serum half-life was reported for unmodified Tz and Tz-based ADCs, T-DM1, of 5.83 days and 4 days, respectively^33^. Both TCO-functionalized and control pre-targeting Tz components exhibited a relatively short circulatory half-life in the plasma in our studies. While the Tz conjugation strategy did not saturate the available amino groups in the antibody, possible changes in the lipophilicity and surface charge of the component would depend on the DOF and the nature of the conjugation groups^34^ and might affect the clearance and circulation time of the antibody. In addition, the nude mice used in our study have a functional complement system and mature B-cells. Therefore, humanized trastuzumab antibodies can be recognized and cleared by the host.

The *in vivo* cell surface labeling, cluster formation, and internalization were assessed using intravital multiphoton imaging, which is a powerful technique with which to study dynamic processes in living animals^35^. This technique is particularly appropriate when observing cancer cells, the tumor microenvironment, and the microvascular architecture in tumor models *in vivo*^36^. Traditional dorsal window chambers are a common accessory used for intravital *in vivo* imaging studies due to the convenience of focusing and choosing a suitable FOV. However, these chambers allow a narrow time-window for tumor growth and imaging, and create an artificial microenvironment for the tumors. These chambers can be optimized for imaging in subcutaneous tumor models, but are difficult to use with orthotopic tumor models^37^. Therefore, in this study, we used a custom-made mouse-holder equipped with a compression window plate that can fix orthotopic tumors to allow imaging without motion artifacts. This set-up facilitates intravital imaging of cancer cells, the tumor microenvironment, and the vascular architecture after a minimally invasive skin-flap surgery (Fig. 4A). This imaging system was used for the real-time visualization of perfusion and extravasation of drug delivery components in the tumor microenvironment. Intravital imaging demonstrated high uptake and internalization of drug-loaded components in the delivery system driven by bioorthogonal click chemistry. After approximately two hours of skin-flap surgery, the rate of the blood flow in the tumor site was reduced, possibly due to the local thrombosis in the area.

Mice in the click-treated group showed a higher therapeutic response, likely due to the tumor uptake of drug-loaded nanocarriers by cluster formation and subsequent cellular internalization (Fig. 5A). The mock-treated delivery system included non-reactive variants of both components and did not facilitate tumor uptake, cluster formation, and internalization driven by bioorthogonal click chemistry. However, we did detect an improved therapeutic response in mock-treated mice compared to untreated controls, and this observation was presumably attributable to the combination of nonspecific accumulation of the cytotoxic nanocarrier through the EPR effect and the therapeutic effect of Tz. The therapeutic efficacy in the click-treated group was significantly higher than the treatment effect in the untreated group (after day 8 onward) or in the mock-treated group (after day 12 onward). At the end of the therapeutic study, the click-treated group showed significantly low tumor sizes compared to both control groups (Fig. 5B). The Kaplan-Meier curves based on the time taken by tumors to reach four times the initial size (Fig. 5C) demonstrated a significant effect of the targeted therapy compared to mock-treated and untreated controls. The typical appearance of tumors that shrank in the click-treated group (left) compared to the mock-treated group (right) is shown in Fig. 5D. The nanoclusters of pre-targeting and drug-loaded delivery components were rapidly internalized by tumor cells through a receptor-mediated endocytosis and led to controlled-release of paclitaxel by enzymatic or acidic cleavage of the linker. During the course of the treatment, we observed no significant change in body weights in any group of mice. The analysis of *in vivo* images also confirmed the liver and kidney uptake of a trace amount of pre-targeting components and a medium amount of drug-loaded nanocarriers at the eight hours post-injection, with no toxicological effects. The liver and kidneys are major sites for metabolism and excretion in the animal body. Drug-induced toxicity in the liver and kidneys could disrupt their functionality and alter the composition of platelets (PLT), lactate dehydrogenase (LDH), blood urea nitrogen (BUN), alanine transaminase (ALT), and aspartate aminotransferase (AST) in the blood. During the treatment in this study, no significant drug-induced toxicity was detected in long liver and kidney blood panels, even at treatments with 3× and 5× doses.

Pre-targeted therapy was originally proposed for radioimmunotherapy using biotin-avidin conjugation chemistry^38^,^39^,^40^,^41^,^42^. A pre-targeting approach was also used for molecular imaging of cancer using specific antibodies and a biotin-avidin *in situ* multi-step conjugation^43^. Other investigators reported a pre-targeting approach using antibodies and bioorthogonal chemistry for imaging and radioimmunotherapy^44^,^45^,^46^. Compared to those previously published reports, our strategy uses a unique combination of specific pre-targeting and cluster formation by multiple click reactions between the pre-targeting and therapeutic carrier components to achieve rapid internalization and delivery of the therapeutic cargo to cancer cells. We also suggest that the cross-linking between neighboring receptors is only plausible on HER2-overexpressing tumor cells; hence, the cluster formation and internalization of drug-loaded nanocarriers are likely not possible in healthy cells. In this approach, the size, location, and stage of tumor can be determined by imaging of the pre-targeting component before the administration of the drug-loaded delivery component. Since trastuzumab is used in this delivery system only as the target-specific molecule, the efficacy of therapy could be high for trastuzumab-resistant tumors as well. This approach also provides direct delivery of therapeutics via HER2-mediated endocytosis, and thus, avoids multi-drug resistance (MDR) transporters^47^. The low pH of subcellular compartments and the enzymatic environment of the cytoplasm trigger the hydrolysis of drug-linkers and controlled-release of Px.

In summary, we have developed a two-step, two-component drug delivery system driven by bioorthogonal click chemistry and evaluated it in preclinical systems, *in vivo*. In this strategy, a trastuzumab-based first component was used to pre-label the HER2(+) tumor cells and a paclitaxel-loaded albumin carrier was subsequently delivered as the cytotoxic treatment component. This new two-component delivery system showed high accumulation of delivery components in the cancer cells and enhanced therapeutic efficacy. Image guidance can be provided by labeling the components with appropriate imaging agents, and can be used for cancer staging (location, size, and HER2 status) by tracking the low/non toxic pre-targeting component with noninvasive imaging. The results of pre-labeling can be used to make clinical decisions regarding the administration and timing of the cytotoxic drug carrier component with suitable chemotherapeutics to maximize efficacy and minimize non-specific toxicity and side effects.

## Materials & Methods

### Biotherapeutics, chemotherapeutics, and chemicals

Trastuzumab was kindly provided by Robert Ivkov, PhD (The Johns Hopkins University School of Medicine), and was used after purification. Paclitaxel was purchased from LKT Laboratories, Inc. The amine-reactive TCO-NHS ester and Tetrazine-Peg_4_-NHS ester were purchased from Sigma-Aldrich Corp, and Kerafast, Inc., respectively. NHS-Alexa Fluor^®^ 488 was purchased from Invitrogen Corp. Dry or HPLC-grade solvents were purchased from Sigma-Aldrich Corp. and used without further purification. XenoLight CF 680 and CF 750 NIR fluorescent dyes were purchased from PerkinElmer, Inc. NHS-Rhodamine and NHS-DyLight 800 dyes were purchased from Life Technologies Corp.

### Formulation of components

Trastuzumab (500 *μ*L of 10 mg/mL in PBS) was treated with TCO-NHS ester (300 moles equiv. in 10–20 *μ*L of dry DMSO) by gently stirring for one hour. Samples were purified by ultracentrifugation followed by HPLC. The resulting Tz(TCO)_6_ (500 *μ*L of 10 mg/mL in PBS) was treated with NHS-AlexaFluor 488, NHS-Rhodamine or NHS-CF-680 (10 moles equiv. in 10 *μ*L of dry DMSO) and stirred for one hour. The degree of labeling (DOL) of the fluorophore was maintained at 2–4. The products were purified by ultracentrifugation followed by HPLC. The resultant Tz(TCO)_6_(AF-488)_4_, Tz(TCO)_6_(Rhod)_2_ and Tz(TCO)_6_(CF-680)_2_ were used as the pre-labeling component *in vitro* confocal fluorescence imaging, intravital multiphoton confocal imaging, *in vivo* imaging and therapeutic studies, respectively (Supplementary Figure S7A). For imaging and therapeutic experiments, albumin or drug-loaded albumin^21^ (2 mL of 10 mg/mL in PBS) was treated with NHS-Peg_4_-Tt (25 moles equiv. 4.0 mg in 20 *μ*L of dry DMSO) and gently stirred for one hour (Supplementary Figure S7B). Modified albumin was labeled with fluorophores by treating with NHS-Rhodamine, NHS-AlxaFluor 488, DyLight 800, or NHS-CF-750 (10 moles equiv. in 10 *μ*L of dry DMSO). The final products, Alb(Peg_4_-Tt)_15_(Rhod)_4_, Alb(Peg_4_-Tt)_15_(AF-488)_2_, Alb(Px)_2.6_(Peg_4_-Tt)_15_DL-800)_2_, and Alb(Px)_2.6_(Peg_4_-Tt)_15_(CF-750)_2_, were used for *in vitro* confocal fluorescence imaging, intravital multiphoton confocal imaging, *in vivo* imaging and image-guided drug delivery studies, respectively. The molecular masses of intermediates at each step were measured by MALDI-TOF (Supplementary Figure S7C). The products were purified by ultracentrifugation followed by HPLC. For therapeutic experiments, albumin was first conjugated with paclitaxel, followed by functionalization with Peg_4_-Tt and labeling with CF-750 according to the procedure described above.

### Ultracentrifugation and HPLC purification

Amicon ultra centrifugation filter units (0.5 mL, 3 kDa, and 15 mL, 30 kDa) were used to concentrate samples and to remove the unreacted low-molecular weight reagents after each step of the conjugation reactions. The samples were further purified by a Waters binary pump/dual absorbance HPLC system equipped with a YMC-Pack Diol-300 (300 × 8.0 mm I.D.; particle size, 5 *μ*m; pore size, 30 nm) size exclusion column, using 0.1 M PBS with 0.2 M NaCl (pH 7.2) as the mobile phase.

### MALDI of components

The molecular weights of modified proteins were determined by MALDI-TOF (Mass Spectrometry and Proteomics Facility, The Johns Hopkins University School of Medicine). The DOF of the functional group and the DOC of paclitaxel were calculated based on the change in molecular weights (Supplementary Figure S7C). The DOL of fluorophores was determined following manufacturers’ protocols.

### Cell lines

The HER2-overexpressing BT-474 cell line was purchased from the American Type Culture Collection (ATCC). The cells were grown in 46-X medium supplemented with 10% FBS and 1% penicillin-streptomycin according to the manufacturer’s protocol, and maintained at 5% CO_2_ in a humidified incubator at 37 °C. Cells were confirmed to be free of mycoplasma infection.

### Human breast cancer mouse models

A pellet of 17*β*-estradiol (0.72 mg/90 day release, Innovative Research of America) was implanted in the subdermal space of each healthy, four-to-six-week-old, female Nu/Nu mouse. After approximately 24 h, BT-474 cells at 70–80% confluency, in fresh medium for <24 h, were collected and prepared with 5 × 10^6^ in 50 *μ*L of 46-X medium:Matrigel (1:1) for each inoculation and maintained at 4 °C. Cells were orthotopically inoculated into the 2^nd^ mammary fat-pads. When tumor volume reached 100–150 mm^3^, mice were used for *in vivo* and intravital imaging and therapeutic experiments. At the end of the experiments, mice were euthanized according to the protocol. All animal experiments were carried out in accordance with protocols approved by the Johns Hopkins University Animal Care and Use Committee, and were conducted in strict compliance with all federal and institutional guidelines.

### *In vitro* confocal fluorescence imaging

BT-474 cells at the third or fourth passage (5 × 10^5^ cells/well in 0.5 mL of 46X medium) were placed in four-well chamber slides and grown for 24–48 h to 70–80 confluency. Cells were first incubated with Tz(TCO)_6_(AF-488)_4_ (20 μg/mL, 130 nM) in PBS+ (PBS supplemented with 0.5% BSA) at room temperature for 20 min. Pre-labeled cells were treated with reactive Alb(Peg_4_-Tt)_15_(Rhod)_4_ or non-reactive Alb(Rhod)_4_ and incubated at room temperature for 15 min. Treated cells were then incubated in fresh 46-X medium for 3 h at room temperature, or 37 °C, and fixed by 4% PFA in PBS. Cells were counterstained by Hoechst 33342 (1 μg/mL in dH_2_O) and wet-mounted for confocal microscopic imaging on a Zeiss Axiovert 200 system equipped with an LSM 510-Meta confocal module.

### *In vivo* Xenogen optical imaging

Mice were injected intravenously with either Tz(TCO)_6_(CF-680)_2_ or Tz(CF-680)_2_ (0.2 mg in 200 *μ*L of sterile PBS), and imaged using a Xenogen IVIS 200 Optical Imaging system at 5, 15, 30 min, 1, 2, 4, and 8 h post-injection. At 8–10 h post-injection time, there were significant amounts of tumor uptake in the pre-targeting components, while the targeting components were below the detection limit in the systemic circulation. At this time point, mice were injected with a drug-loaded nanocarrier, Alb(Px)_2.6_(Peg_4_-Tt)_15_(DL-800)_2_ (2.0 mg in 200 *μ*L of sterile PBS), and we continued to image them to see the tumor uptake of the delivery component.

### Minimally invasive surgery and intravital imaging

Mice were injected intravenously with Tz(TCO)_6_(Rhod)_2_ (0.2 mg in 200 *μ*L of sterile PBS). After 8 h, mice were anesthetized with ketamine and acepromazine (i.p. ketamine 100 *μ*g/g body weight; acepromazine 10 *μ*g/g body weight) and placed on the custom-made mouse-holder in the supine position (Fig. 4A). The holder was maintained at 37 °C using a feedback-controlled heating pad. The tumor was disinfected using alcohol-prep and the dorsolateral skin of the tumor was pulled and cut using a forceps and micro surgical scissors. The “U”-shaped skin-flap, with a ~0.5 cm width, was immobilized using stitches. The skin opening treated with saline was covered with a compression plate and imaged through the window on the compression plate, which was parallel to the longitudinal axis. Intravital images were obtained under 10× or 25× magnification using an Olympus FV1000MPE multiphoton laser-scanning microscope. The drug-loaded nanocarrier, Alb(Peg_4_-Tt)_15_(AF-488)_2_ or control Alb(AF-488)_2_ (2.0 mg in 200 *μ*L of sterile PBS), was injected through a catheter secured to the tail vein and was continuously observed in real-time.

### Therapeutic procedure

Three groups of female Nu/Nu mice, orthotopically inoculated with BT-474 human breast cancer cells, were administered Tz(TCO)_6_(CF-680)_2_, Tz(CF-680)_2_ (both intravenously at a dose of 10 mg/kg) or saline, and were considered click-treated, mock-treated control, and untreated control groups (n = 5 each group), respectively. After 12 h, mice in the click-treated and mock-treated groups were administered Alb(Px)_2.6_(Peg_4_-Tt)_15_(CF-750)_2_ at a dose of 25 mg/kg, while mice in the untreated group were given saline as a control. The second therapeutic dose was given after two weeks. The sizes of the tumors were measured with a caliper every fourth day. The tumor volume was calculated using the formula (*L* × *W*^*2*^)*π*/*6*, where *L* is the longest diameter (the major axis) and *W* is the width dimension, which is perpendicular to the major axis.

### Statistical analysis

The statistical analysis between treated and untreated groups was performed using JMP 12.1.0 Statistical Discovery^TM^ from SAS. The significance of therapeutic effects in each pair was analyzed by the nonparametric, multiple comparisons Wilcoxon each pair test. A p value of less than 0.05 was considered significant (*p < 0.05, **p < 0.005).

## Additional Information

**How to cite this article**: Hapuarachchige, S. *et al*. Bioorthogonal two-component drug delivery in HER2(+) breast cancer mouse models. *Sci. Rep*. **6**, 24298; doi: 10.1038/srep24298 (2016).

## Supplementary Material

Supplementary Information

## Figures and Tables

**Figure 1:**
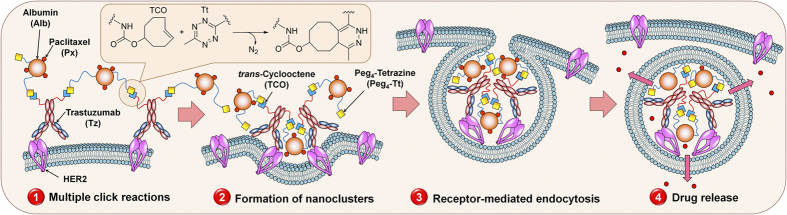
Bioorthogonal two-component, two-step drug delivery system. A model illustrating the strategy of the target-specific, two-component, two-step drug delivery driven by bioorthogonal *trans*-cyclooctene/tetrazine click chemistry in HER2(+) cancer cells.
