# Supplementary material for: Bioorthogonal two-component drug delivery in HER2(+) breast cancer mouse models
Source: Sci Rep. 2016 Apr 12;6:24298. doi: 10.1038/srep24298 (PMC4828666; doi:10.1038/srep24298)
Supplement: Supplementary Information [file srep24298-s1.doc]

*Supplementary information*

**Bioorthogonal two-component drug delivery in HER2(+) breast cancer mouse models.**

Sudath Hapuarachchige, Yoshinori Kato and Dmitri Artemov*

* Corresponding author: dartemo2@jhmi.edu


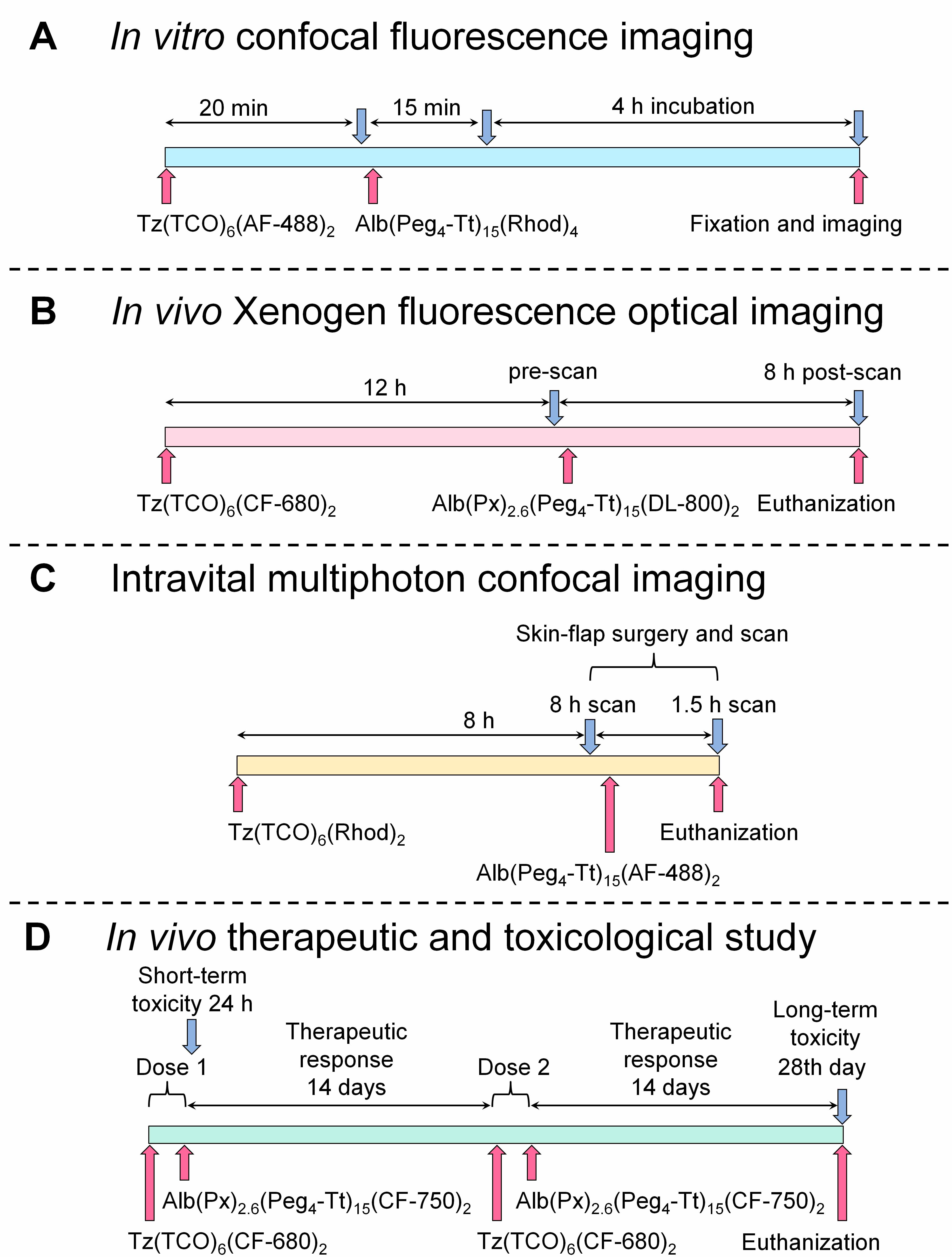


**Figure S1. Treatment schedules for imaging and therapy.** (**A**) *In vitro* confocal fluorescence imaging, (**B**) *in vivo* Xenogen fluorescence optical imaging, (**C**) intravital multiphoton confocal imaging, and (**D**) *in vivo* therapeutic study.


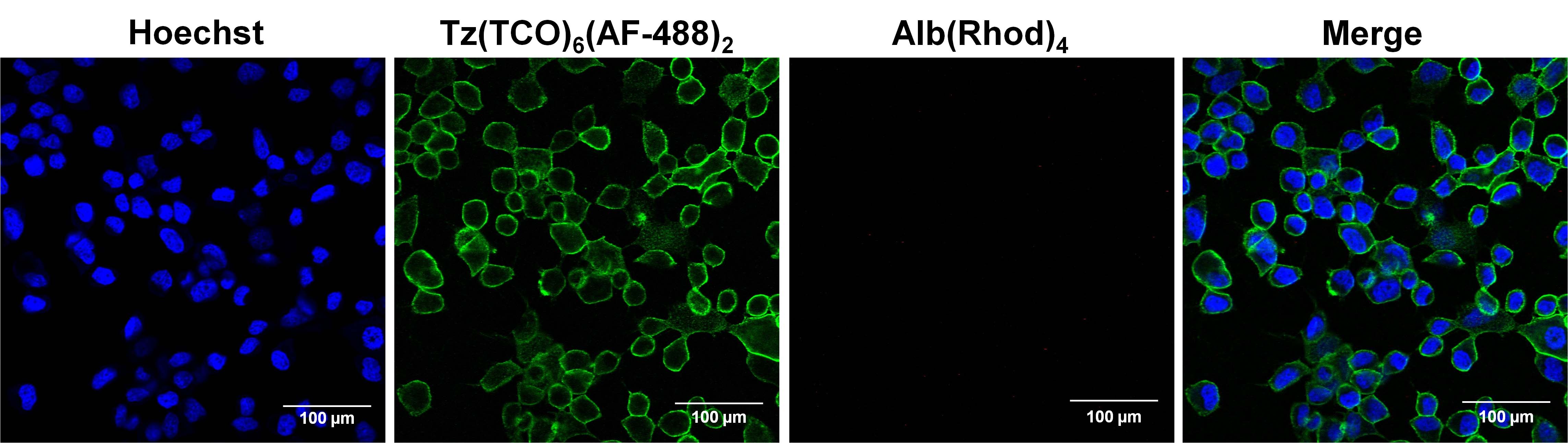


**Figure S2. Control *in vitro* fluorescence imaging of internalization strategy.** BT-474 cells were treated with pre-targeting Tz(TCO)_6_(AF-488)_2_ (green) followed by control Alb(Rhod)_4_ (red) carrier component incubated for 4h at 37 °C (Scale bar: 100 µm). Channels were merged to observe the cell surface labeling, co-localization followed by internalization of the components.


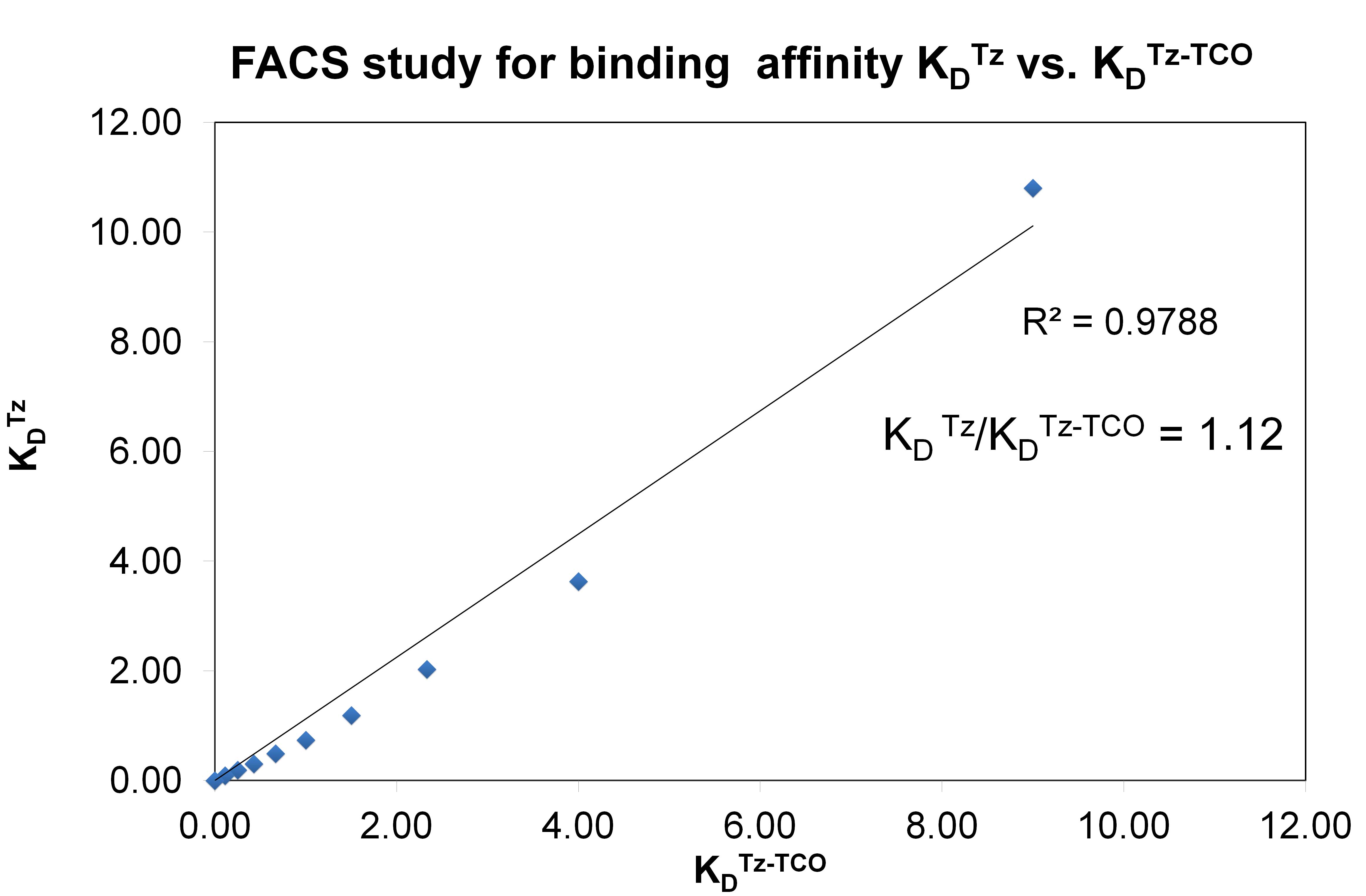


**Figure S3. Comparison of binding affinity of Tz and Tz(TCO)_6_.** Fluorescence of BT-474 cells incubated with increased concentrations of the pre-targeting components Tz and Tz(TCO)_6_ was measured with FACS and results were processed with a linear regression model.


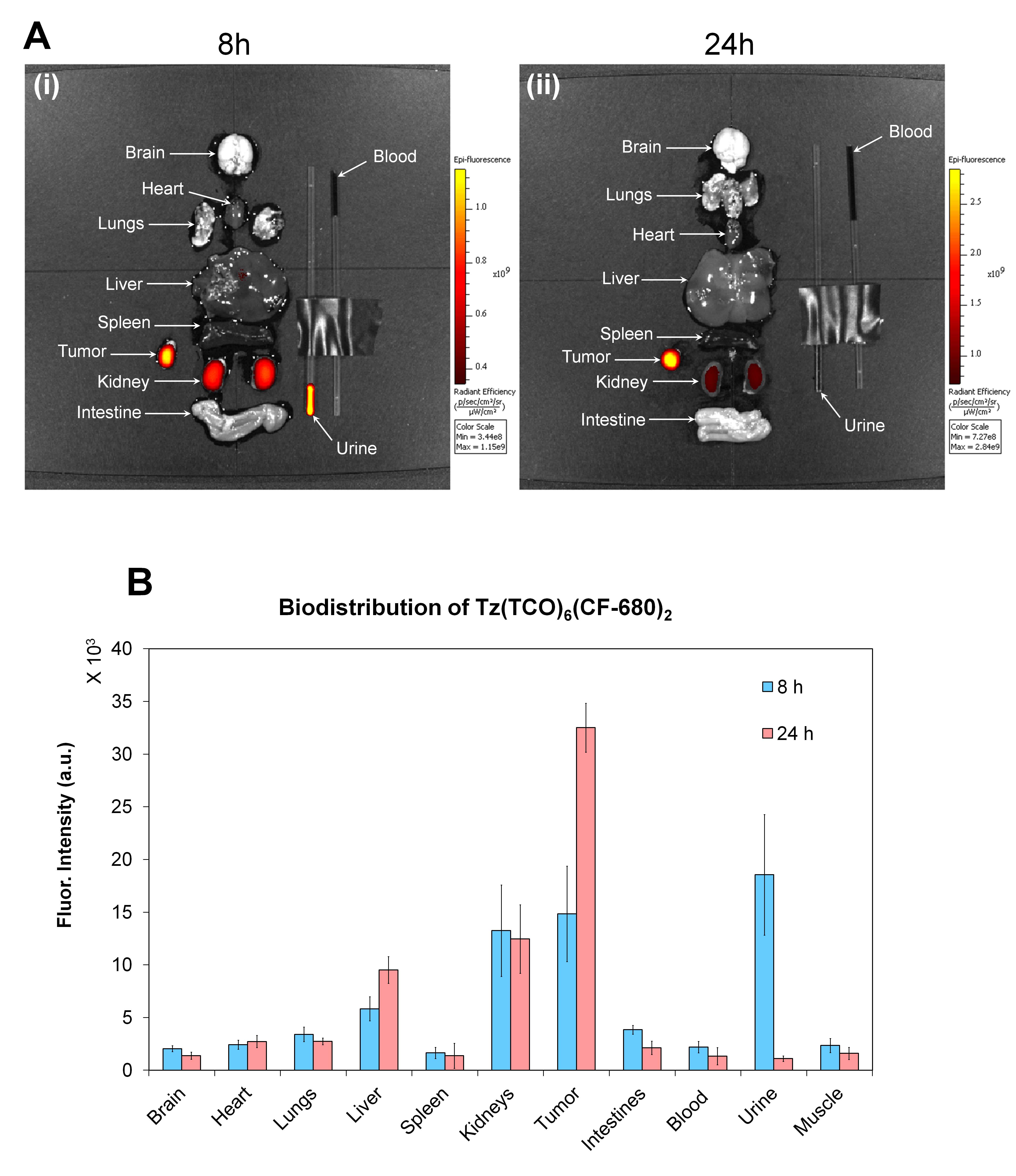


**Figure S4. Biodistribution of Tz(TCO)_6_(CF-680)_2_ in mouse models.** (**A**) Selected organs, blood, urine and tumor were extracted after 8 (i) and 24 (ii) hours and imaged using Xenogen fluorescence optical imaging system. (**B**) Quantitative analysis of Tz(TCO)_6_(CF-680)_2_ biodistribution at 8 and 24 h time points.


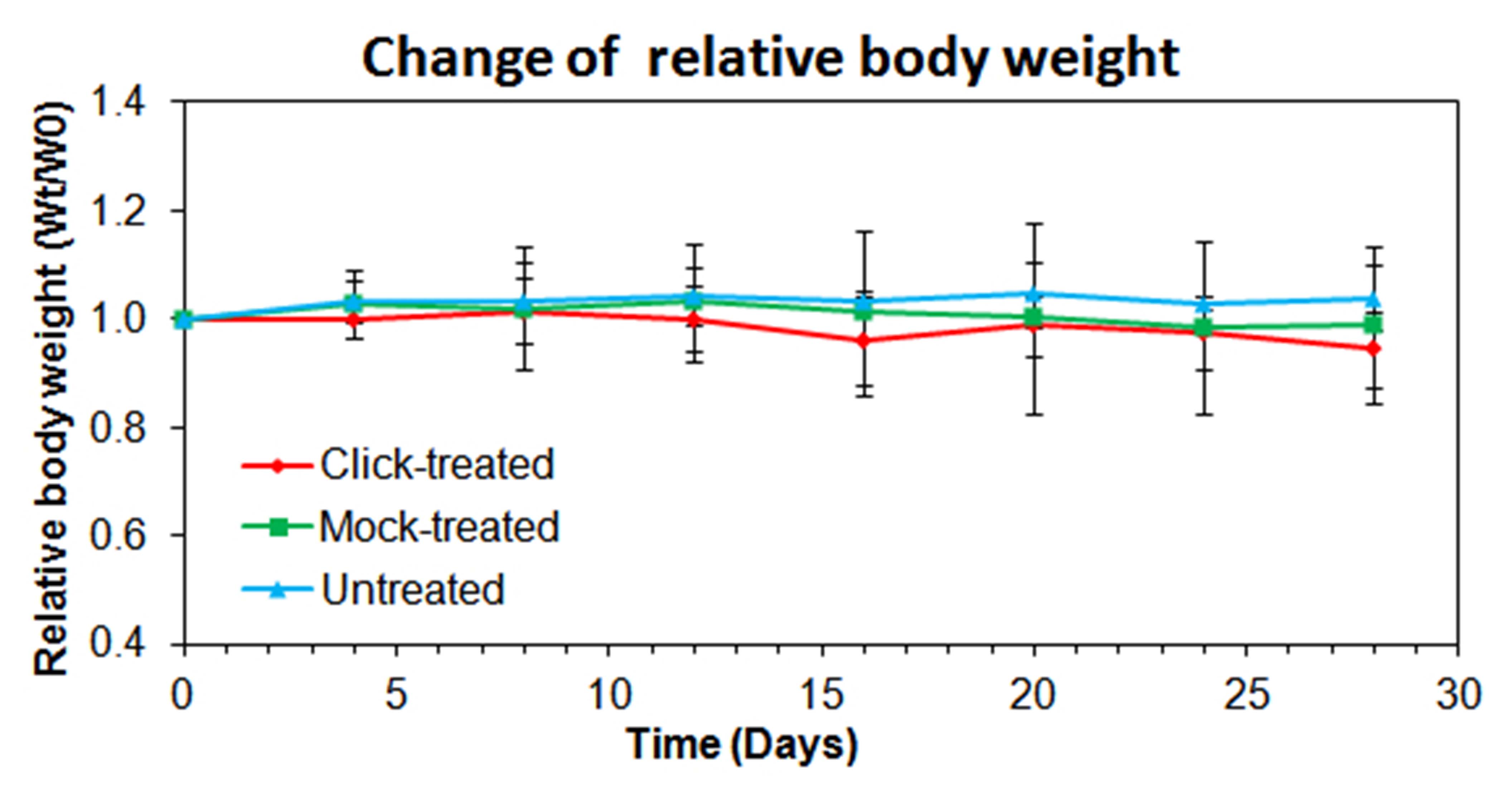


**Figure S5.** Change in relative body weights of mice over the 28 days of treatment period.


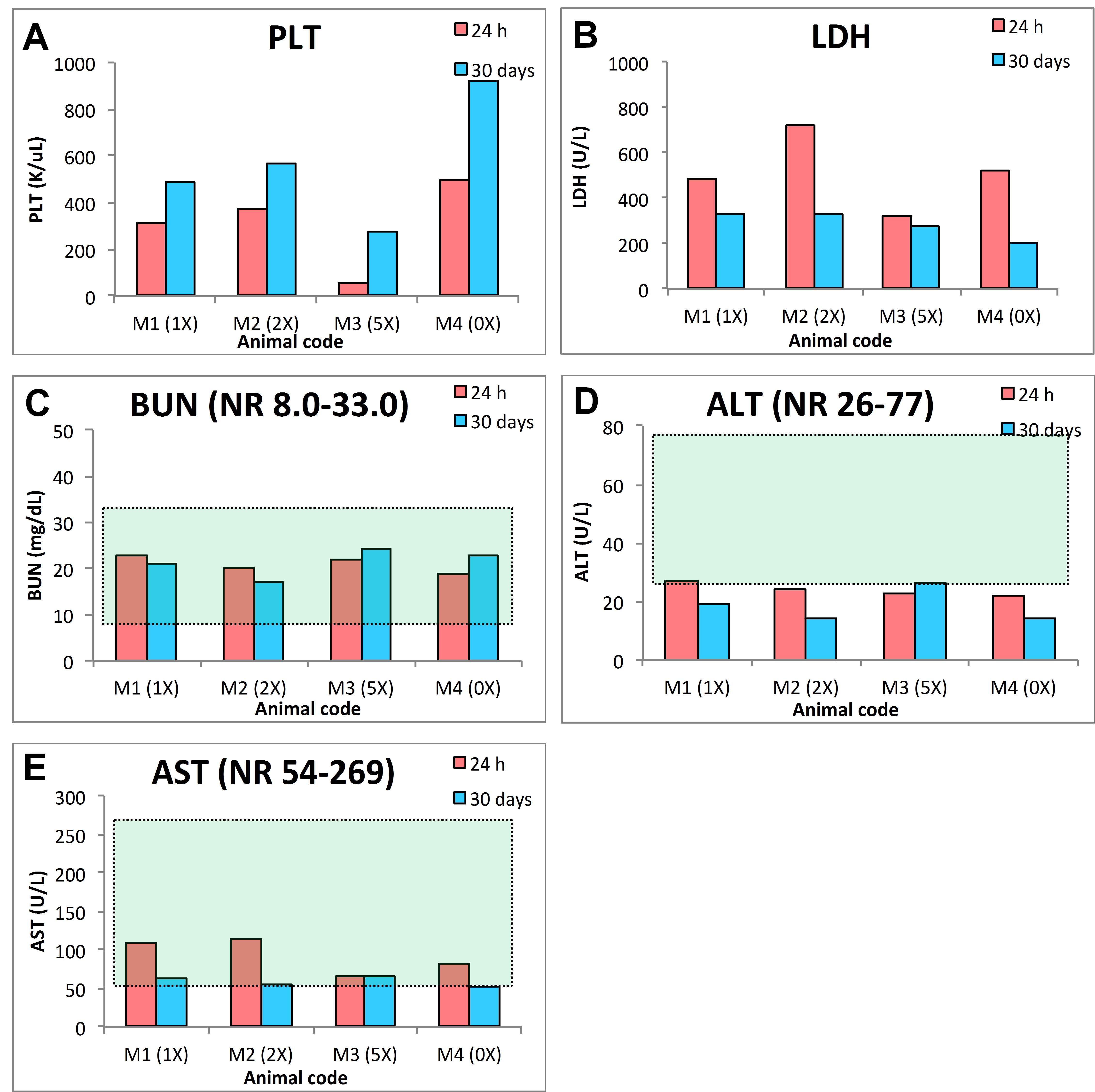


**Figure S6. Representative toxicological studies performed for 24 h and 28 days after the initial administration.** Mice in click-treated and untreated-control groups were received 1x, 2x, and 5x doses of both components on day 0 day and analyzed the blood at 24 h for short-term toxicological effects. The same sets of mice were received second dose on day 14, and the long-term toxicological effects were analyzed on day 28. Untreated-control mice received saline injections during the treatment. (**A**) PLT – platelet concentration; (**B**) LDH – lactic dehydrogenase isoenzymes; (**C**) BUN – blood urea nitrogen; (**D**) ALT – alanine aminotransferase; (**E**) AST – aspartate aminotransferase.


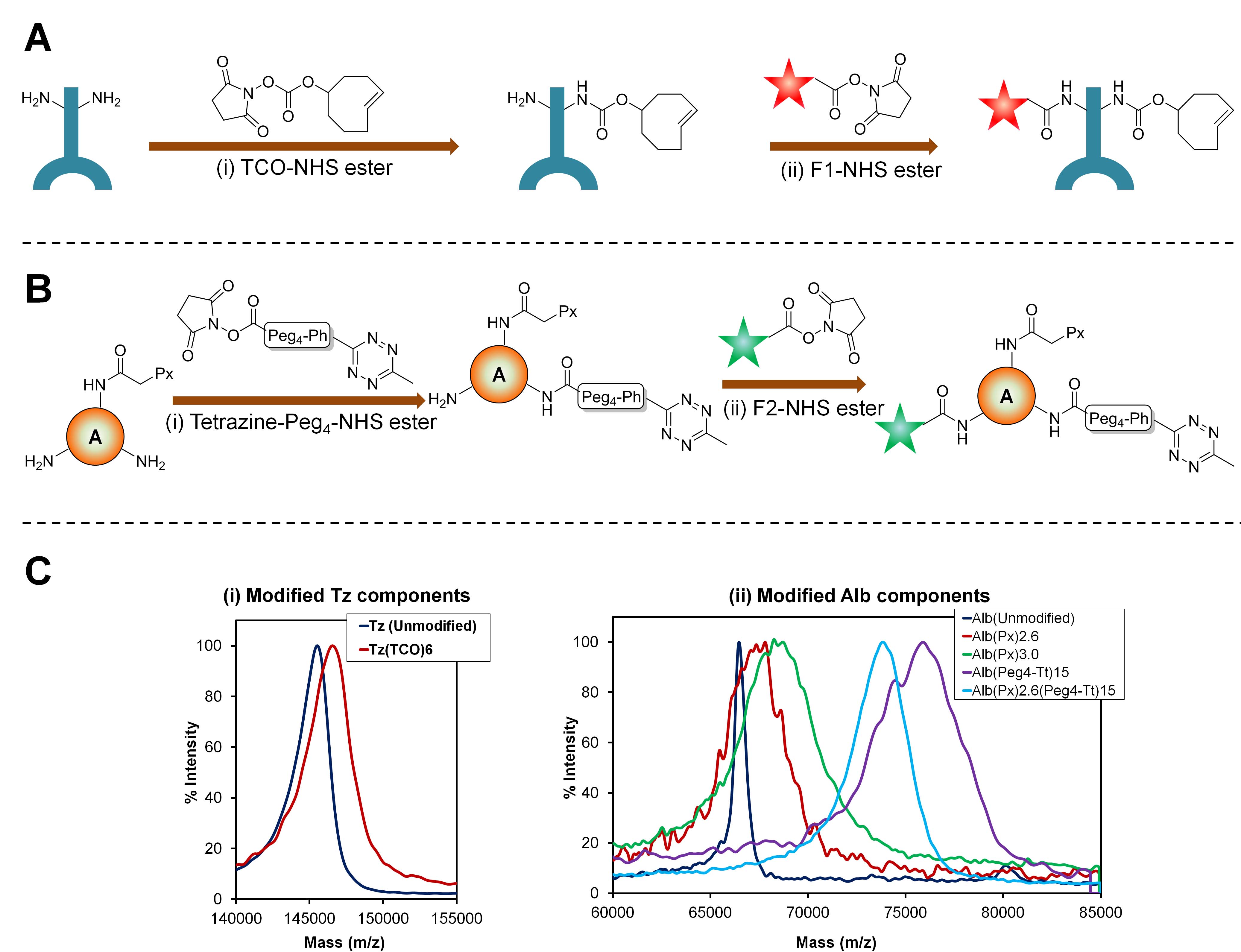


**Figure S7. Formulation of pre-targeting and delivery components.** (**A**) Formulation of Tz based pre-targeting components (i) functionalization of Tz with TCO followed by (ii) labeling with F-1 fluorophore. (**B**) Formulation of Alb based drug-loaded nanocarriers, (i) functionalization of paclitaxel drug-loaded Alb(Px)_2.6_ with Peg_4_-Tt followed by (ii) labeling with F-2 fluorophore. (**C**) MALDI-TOF/MS spectra of components. (i) Change in molecular weights after functionalization of Tz with TCO groups. (ii) Change in molecular weights of Alb after conjugation with Px followed by Peg_4_-Tt functionalization.
